# Essential Medicine

**Published:** 1982

**Authors:** 


### Book Review

**ESSENTIAL MEDICINE**
**J. L. Burton**
**Churchill Livingstone 1981**
**(pp.157, £4.25)**

This paper back is clearly written, mostly in note form, and well illustrated with diagrams. It contains sufficient material for nurses, physiotherapists and other para-medical professions, and as the author states, for medical students on their first firm. I would like to have seen more emphasis on psycho-somatic and emotional disorders which are so common in medical ward patients. One could criticise the lack of balance e.g. twice as much space on congenital hearts as on rheumatoid disease, almost as much on rickets and osteomalacia as on strokes, a page on polio and neurosyphilis but no mention of the common tension headaches. In therapeutics, cimetidine, carbamazepine, valproate and vasodilators for hypertension are missing, and ergotamine is the only recommended treatment for migraine. In a book of this size, one doubts the need for a whole page diagram on portacaval shunt, or on different sizes of spleen. Inevitably when much of the text is in the form of notes, correct emphasis is difficult as for example in the section on epilepsy where idiopathic causes are almost mentioned as an afterthought. The chapter on drugs could be expanded, and encouragement given for the reader to refer to the British National Formulary.

Despite these minor criticisms, this book written by a local author, will serve a useful role.

